# Increased BBB Permeability Enhances Activation of Microglia and Exacerbates Loss of Dendritic Spines After Transient Global Cerebral Ischemia

**DOI:** 10.3389/fncel.2018.00236

**Published:** 2018-08-03

**Authors:** Furong Ju, Yanli Ran, Lirui Zhu, Xiaofeng Cheng, Hao Gao, Xiaoxia Xi, Zhanli Yang, Shengxiang Zhang

**Affiliations:** ^1^Gansu Key Laboratory of Biomonitoring and Bioremediation for Environmental Pollution, School of Life Sciences, Lanzhou University, Lanzhou, China; ^2^School of Basic Medical Sciences, Lanzhou University, Lanzhou, China

**Keywords:** BBB, ischemia, reperfusion, microglia, spine, mannitol

## Abstract

Ischemic stroke can induce rapid disruption of blood-brain barrier (BBB). It has been suggested that increased BBB permeability can affect the pathological progression of ischemic tissue. However, the impact of increased BBB permeability on microglial activation and synaptic structures following reperfusion after ischemia remains unclear. In this study, we investigated microglial activation, dendritic damage and plasticity of dendritic spines after increasing BBB permeability following transient global cerebral ischemia in the somatosensory cortices in mice. Bilateral common carotid artery ligation (BCAL) was used to induce transient global cerebral ischemia. Mannitol was used to increase the BBB permeability. Intravital two-photon imaging was performed to image the dendritic structures and BBB extravasation. Microglial morphology was quantitated using a skeletonization analysis method. To evaluate inflammation of cerebral cortex, the mRNA expression levels of integrin alpha M *(CD11b), CD68*, chemokine (C-X-C motif) ligand 10 *(IP10)* and tumor necrosis factor alpha *(TNF-α)* were measured by fluorescent quantitative PCR. Intravital two-photon imaging revealed that mannitol caused a drastic increase in BBB extravasation during reperfusion after transient global ischemia. Increased BBB permeability induced by mannitol had no significant effect on inflammation and dendritic spines in healthy mice but triggered a marked de-ramification of microglia; importantly, in ischemic animals, mannitol accelerated de-ramification of microglia and aggravated inflammation at 3 h but not at 3 days following reperfusion after ischemia. Although mannitol did not cause significant change in the percentage of blebbed dendrites and did not affect the reversible recovery of the dendritic structures, excessive extravasation was accompanied with significant decrease in spine formation and increase in spine elimination during reperfusion in ischemic mice. These findings suggest that increased BBB permeability induced by mannitol can lead to acute activation of microglia and cause excessive loss of dendritic spines after transient global cerebral ischemia.

## Introduction

Under normal physiological conditions, only specific substances from the bloodstream are allowed to enter the brain parenchyma due to a highly specialized blood-brain barrier (BBB; Betsholtz, 2014; Zhao et al., 2015). BBB can restrict the entry of peripheral inflammatory cytokines, hemoglobin, albumin and neurotoxic factors into the cerebral parenchyma (Zlokovic, 2008; Bell et al., 2010), and plays an essential role in maintaining the homeostasis of central nervous system (CNS) microenvironment (Hawkins and Davis, 2005). However, the integrity of BBB is vulnerable to pathological insults and can be altered in many CNS diseases, including stroke, Alzheimer’s disease (AD), Parkinson’s disease and acute traumatic brain injury (Hawkins and Davis, 2005; Ulrich et al., 2015; Nahirney et al., 2016; Olmedo-Diaz et al., 2017; Zhang et al., 2017). Among these diseases, stroke can rapidly cause disruption of BBB. Severe stroke can lead to blood vessel damage and drastic BBB leakage within hours or less after stroke onset, and even transient ischemia can induce marked extravasation (Nishimura et al., 2006; Zhang and Murphy, 2007; Zhu et al., 2017b). The restoration of the disrupted BBB takes hours or even days depending on the duration and severity of stroke. The disrupted BBB induced by transient ischemia can be restored within hours (Zhu et al., 2017b), but the prolonged increase in vascular permeability can still be detected from day 3 to day 21 after middle cerebral artery occlusion (MCAO; Lin et al., 2008).

Some studies suggest that the disrupted BBB after stroke may have detrimental effect on the cerebrum, which includes the loss of cerebral blood-pressure autoregulation (Sandoval and Witt, 2008), the permeation of the immune cells which release inducible NO synthetase into the infarct/peri-infarct region (Moro et al., 2004), and the inflammatory response that further propagates the immune response (Gelderblom et al., 2009). In addition, previous studies indicate that early BBB disruption is the cause rather than the result of parenchymal cell injury in ischemic stroke (Shi et al., 2016), and aggravated cerebral edema is one of the consequences of BBB disruption after focal cerebral ischemia in Cav-1-deficient mice (Choi et al., 2016). However, some other studies indicate that there is little acute collateral damage to dendrites by extravasation during the first few hours after the disruption of BBB (Zhang and Murphy, 2007; Zhu et al., 2017b). Thus, the impact of increased BBB permeability on brain tissue after stroke remains unclear. In recent years, BBB permeation-aided stem cell therapy has shown great potential in the acute stage of stroke (Ishikawa et al., 2013; Chen et al., 2016). The lack of available therapies and the devastating effects of ischemic stroke impel many researchers to focus on enhancing BBB permeability to apply macromolecular drugs or stem cells to ischemic brain (Borlongan et al., 2012). However, the risk of increased BBB permeability and its impact on the activation of microglia and spine dynamics after ischemic stroke remains unknown.

In the present study, mannitol was used to increase the BBB permeability, and a reversible global cerebral ischemic model of mice combined with confocal and two-photon microscopy was used to evaluate the effect of increased BBB permeability on both the microglial response and restoration of dendritic structures of layer 5 pyramidal neurons in somatosensory cortex. Our results showed that although mannitol-induced de-ramification of microglia and inflammatory response only at early stage during reperfusion after ischemia, it induced a significant loss of dendritic spines when layered on top of ischemia. These results suggest that increased BBB permeability is detrimental to synaptic structures after ischemia and regulating BBB permeability could provide a new strategy to treat ischemic stroke.

## Materials and Methods

### Animals

Transgenic mice (Thy1-YFP line H, JAX #003782) expressing yellow fluorescent proteins (YFP) in the layer 5 pyramidal neurons, and CX3CR1^GFP/+^ mice expressing green fluorescent proteins (GFP) in the microglia were both purchased from the Jackson Laboratory. All the animals were maintained in a C57/BL6 background and bred in the Laboratory Centre for Basic Medical Sciences, Lanzhou University. Mice had free access to food and clean water with a regular cycle of light in 24 h (12-h light/12-h dark cycle) at 22 ± 2°C. Transgenic mice of both sexes aged 3–5 months and weighing 20–30 g were used in this study. This study was carried out in accordance with the regulations of Lanzhou University and the ARRIVE guidelines. All experimental procedures and protocols were approved by the Ethics Committee of Lanzhou University.

### Global Cerebral Ischemia Model

Bilateral common carotid artery ligation (BCAL) was used to induce global ischemia as described in previous study (Zhu et al., 2017a). Briefly, each animal was deeply anesthetized with the mixture of ketamine (150 mg/kg body weight) and xylazine (25 mg/kg body weight) through intraperitoneal injection, and then a thinned-skull cranial window above somatosensory cortex was made by using a high-speed dental drill. After that, animals were laid in supine position and the common carotid arteries were exposed and encircled with surgical sutures. The mice were subjected to 30 min of transient ischemia at room temperature by tightening the surgical sutures. To minimize the variation of cortical ischemia, the reduction of blood flow and the beaded structure of dendrites were confirmed through cranial window by intravital two-photon imaging, and induction of global ischemia was considered to be successful only when beaded dendrites were observed within 10 min after occluding the common carotid arteries. After 30 min of bilateral common carotid arteries occlusion, surgical sutures were untied to re-perfuse the blood vessels. The animals were placed back to the heating pad after BCAL before fully awakening to support the recovery and enhance the survival rate.

### Administration of Mannitol

To open BBB, 20% mannitol (5 ml/kg) was administered through tail vein injection in mice. For ischemic mice, saline or mannitol was injected for three times at an hourly interval via tail vein during reperfusion after 30 min of ischemia. For the sham-operated mice, saline or mannitol was injected at an hourly interval for three times after 30 min following sham-operation. The mice were divided into four groups: sham-control group (sham-operated and saline-injected mice), sham-mannitol group (sham-operated mice injected with mannitol), BCAL-control group (mice subjected to BCAL and injected with saline) and BCAL-mannitol group (mice subjected to BCAL and injected with mannitol).

### Two-Photon and Confocal Imaging

Intravital two-photon imaging was performed as described in previous studies (Yang et al., 2010; Zhu et al., 2017b). Briefly, the mouse was deeply anesthetized, and body temperature was kept at 37 ± 0.5°C by a heating pad, then the skull was exposed and tightly glued to a custom-made metal frame and then fixed to a steel plate. A 2 × 2 mm^2^ area of skull with the center position at −1.5 mm from bregma and 2.0 mm from midline was thinned to ~25 μm by using a high-speed dental drill. Real-time two-photon images of dendritic structures were acquired at zoom 4 through water-immersion objective lens (×25/1.05; Olympus). The laser was adjusted to 920 nm and the Z step size was set to 0.75 μm. Repeated Z-series of 60–100 optical sections (1024 × 1024 pixel arrays) were collected at fixed time points. For cell density and skeletonization analysis, confocal images of 30–60 μm sections were obtained with an Olympus confocal microscope at a 1 μm interval. All images were analyzed by using ImageJ software^1^.

### Microglial Skeletonization Analysis

All images of microglia were collected from somatosensory cortex. Skeleton images of microglia from fixed tissue of CX3CR1^GFP/+^ mice were analyzed by ImageJ software as described before (Morrison and Filosa, 2013). Briefly, a macros plugin (“Plugin” > “Process” > “Smooth 3D”; radius, 0.3–0.8) was used to process the images. Analytic skeleton plugin^2^ was applied to collected data to quantitate the total number of microglial process endpoints and total length of microglial process, and skeletonization plugin (2D/3D) was used to generate the skeletonized images.

### Quantitative Evaluation of BBB Permeability

Evans blue (EB) was used as the tracer of BBB hyper-permeability, and EB solution (10 mg/ml; 2.5 ml/kg) was injected via tail vein injection. As a reflection of the extravasation of the blood presented in the tissue, the fluorescence intensity of EB was imaged using two-photon microscopy and quantified with ImageJ. To quantify the change of extravasation, we chose the same segment of blood vessels (diameter ≥5 μm) identified from the 3D image stacks at different time points. A region of interest (ROI) with fixed size (5 μm × 5 μm) was selected on a blood vessel and another ROI with the same size next to the blood vessel was chosen to measure the fluorescence intensity of the extravasation. To compensate the variation caused by altered imaging conditions, the fluorescence intensity of blood extravasation was normalized as the percentage of fluorescence intensity of blood vessel. Thirty pairs of ROI were chosen and quantified in each mouse.

### Immunofluorescent Labeling

Brain tissue fixed by 4% paraformaldehyde (PFA) were washed in phosphate buffered saline (PBS), and sectioned (30 μm) on a vibrating microtome (Leica). Sections were blocked with 10% goat serum dissolved in PBS for 30 min and then incubated with NeuN antibodies (1:200, Rabbit, Millipore) diluted in a buffer consisted of 0.01% Triton X-100 and 5% goat serum for 12 h at 4°C. The sections were washed and then incubated with a secondary antibody (1:500, Goat anti-Rabbit TRITC, ZSGB-BIO) at room temperature.

### Real-Time Fluorescence Quantitative PCR Analysis

Mice (CX3CR1^GFP/+^) from all four groups (*n* = 6/group) used for qRT-PCR analysis were from a separate group but also subjected to skull thinning surgery. Cortical tissues were obtained under a dissecting microscope after transcardiac perfusion with PBS. RNA was extracted by RNA Extraction Kit (TaKara) and quantified by Nanodrop (Thermo Scientific). The integrity of RNA was checked via gel (2% agarose) electrophoresis, and reversely transcribed by Reverse Transcription Kit with gDNA Eraser (Applied Biosystems). Expression of genes was analyzed via real-time fluorescence quantitative PCR by using iTaqTM Universal SYBR^®^ Green Supermix (Bio-Rad). The custom designed gene-specific primers (GENEWIZ) are shown as follows: chemokine (C-X-C motif) ligand 10 (IP10; FW, CTGCCGTCATTTTCTGCCTC, RV, TTCAAGCTTCCCTATGGCCC), Integrin alpha M (CD11b; FW, CCAAGACGATCTCAGCATCA, RV, TTCTGGCTTGCTGAATCCTT), CD68 (FW, GGGGCTCTTGGGAACTACAC, RV, GTACCGTCACAACCTCCCTG) and tumor necrosis factor alpha (TNF-α; FW, GACGTGGAACTGGCAGAAGA, RV, ACTGATGAGAGGGAGGCCAT). The glyceraldehyde-3-phosphate dehydrogenase (GAPDH) was chosen as the reference (Olmos-Alonso et al., 2016). The relative quantity of mRNA levels was determined by the process of 2^−ΔΔCT^.

### Data Analysis

Two-photon image analysis was carried out using ImageJ. To quantitate changes of dendritic spines, we collected the same dendritic segments which were carefully identified from the image stacks at different time points, and more than 200 spines selected from the image stacks were counted in each mouse. The percentage of spine elimination was calculated as the number of lost spines/pre-existing number of spines, and the percentage of spine formation was calculated as the number of new spines/pre-existing number of spines. To quantitate the percentage of blebbed dendrites, we used a custom designed macro to mark the dendrites at 20 μm fixed length and to calculate the length of the selected dendritic segments. Percentage of blebbed dendrites was calculated as total length of blebbed dendrites/total length of dendrites. SPSS software was used for all statistical analysis. Analysis of variance and two-tailed unpaired *t*-test were used for comparison between two groups; one-way analysis of variance (ANOVA) was performed for comparison among three or more groups. Results were presented as mean ± standard error of the mean, *p < 0.05 and **p < 0.01.

## Results

### Mannitol-Induced Changes in BBB Permeability During Reperfusion After Transient Global Ischemia

**Figure 1:**
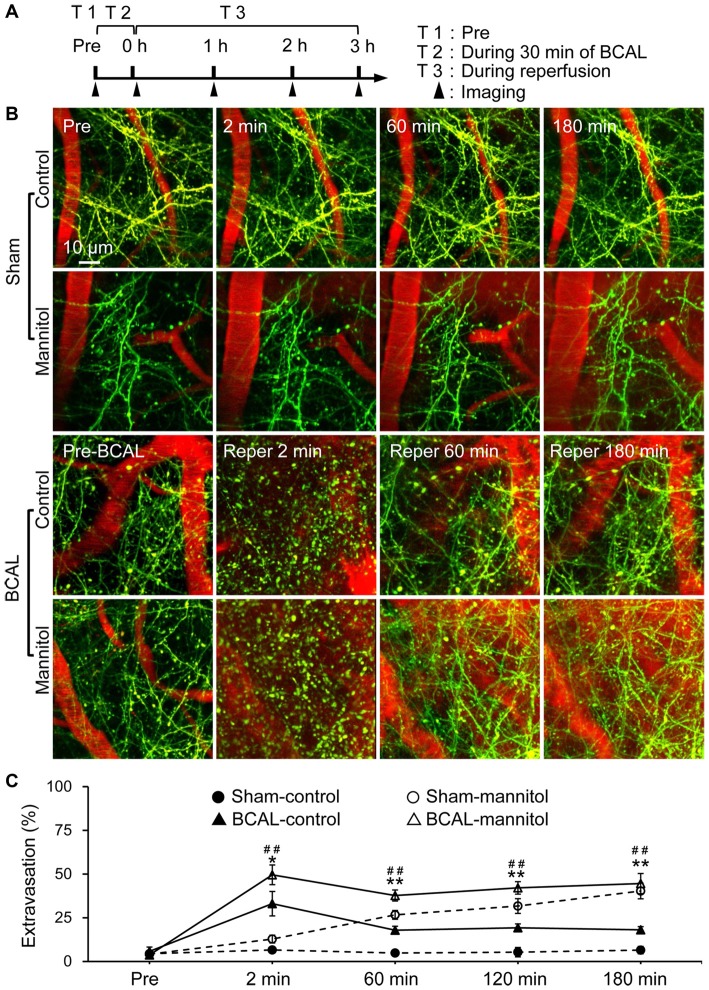
Mannitol-induced change in blood-brain barrier (BBB) permeability after transient global cerebral ischemia. **(A)** Timeline shows time points for imaging during the transient ischemia-reperfusion model. Filled arrowheads show the imaging time points. **(B)** Two-photon images show Evans blue (EB) labeled extravasation (red) and dendritic structures (green) at pre, 2 min, 60 min and 180 min, respectively in sham-control mice, sham-mannitol mice, bilateral common carotid artery ligation (BCAL)-control mice and BCAL-mannitol mice. Note that beaded dendrites induced by transient ischemia restored following reperfusion in both BCAL-control mice and BCAL-mannitol mice. **(C)** Group data shows relative changes of extravasation at different time points (*n* = 6 mice per group. Sham-control vs. Sham-mannitol (*) *p < 0.05 and **p < 0.01; BCAL-control vs. BCAL-mannitol (^#^) ^##^*p* < 0.01).

To alter BBB permeability in cerebral cortex *in vivo*, we established a BBB leakage model by administering 20% mannitol through tail vein. Mannitol is clinically used for hyperosmolar therapy, and can cause an acute increase in BBB permeability (Joshi et al., 2011; Song et al., 2016). We first assessed mannitol-induced BBB leakage in transgenic mice (Thy1-YFP line H) of sham group by EB assay. Intravital transcranial two-photon microscopy was used to image the dendritic structures and extravasation of blood before and after mannitol injection (Figures 1A,B). Extravasation from blood vessels was quantified by densitometry of red fluorescence of EB in brain parenchyma. In sham-control mice, blood vessels were clearly imaged as no significant extravasation of blood (Figures 1B,C). On the contrary, marked extravasation was observed at 2 min in sham-mannitol mice (extravasation (%): sham-control 6.58 ± 1.62% vs. sham-mannitol 12.78 ± 2.24%; *p < 0.05), and a continuous increase in extravasation was found within 180 min after mannitol injection. To further assess the effect of mannitol on BBB permeability after global ischemia, YFP transgenic mice were subjected to 30 min of BCAL, and mannitol or saline was injected immediately after the surgical sutures were loosened. We evaluated the recovery of dendritic structures and BBB leakage at different time points after reperfusion in BCAL-control mice. Our previous studies have shown that ischemia can cause rapid damage to dendritic structures (Zhu et al., 2017b). In present study, live imaging indicated that dendritic structures were still beaded at 2 min after reperfusion in BCAL-control mice. In addition, significant increase in extravasation was observed at this time point (Figures 1B,C, and Supplementary Figure S1A). Repeated imaging showed that the dendritic structures were recovered at 60 min after reperfusion, and the extravasation was significantly declined compared with that at 2 min after reperfusion (Figures 1B,C, and Supplementary Figure S1C; extravasation (%): BCAL-control, reperfusion 2 min 33.06 ± 7.01% vs. reperfusion 60 min 17.86 ± 2.28%; *p < 0.05, respectively). Importantly, with the extended reperfusion time, there were no significant changes in extravasation at both 120 min and 180 min compared with that of 60 min after reperfusion (Figures 1B,C; BCAL-control mice). The recovery of dendritic structures and the change in extravasation were then evaluated in BCAL-mannitol mice after ischemia-reperfusion process. The blood extravasation of BCAL-mannitol mice was significantly increased compared to that of sham-mannitol mice at 2, 60 and 120 min (Figures 1B,C; *p < 0.05). Similar to BCAL-control mice, intravital imaging revealed the dendritic structures in BCAL-mannitol mice were beaded at 2 min after reperfusion following BCAL. However, the extravasation in BCAL-mannitol mice was significantly increased compared with that of BCAL-control mice at 2 min after reperfusion (Figures 1B,C, and Supplementary Figure S1A; *p < 0.05). Repeated imaging showed that dendritic structures were recovered at 60 min after reperfusion in the BCAL-mannitol mice. The extravasation (relative to the fluorescence intensity of blood vessel) was significantly declined at 60 min compared to 2 min in BCAL-mannitol mice but was still significantly higher than that in BCAL-control mice at 60 min after reperfusion (Figures 1B,C, and Supplementary Figure S1C; extravasation (%): 60 min, BCAL-control 17.86 ± 2.28% vs. BCAL-mannitol 37.72 ± 3.16%; **p < 0.01). In addition, the extravasation of the BCAL-mannitol mice did not significantly increase at 120 min and 180 min compared to that at 60 min after reperfusion but was significantly higher than that in BCAL-control mice during reperfusion (Figures 1B,C extravasation (%): 120 min, BCAL-control 19.25 ± 2.24% vs. BCAL-mannitol 42.09 ± 3.57%; **p < 0.01; 180 min, BCAL-control 18.11 ± 1.80% vs. BCAL-mannitol 44.55 ± 5.76%; **p < 0.01). These results demonstrated that mannitol enhanced BBB permeability and caused excessive extravasation during reperfusion in ischemic mice.

### Effect of Compromised BBB on Microglial Morphology and Inflammation

**Figure 2:**
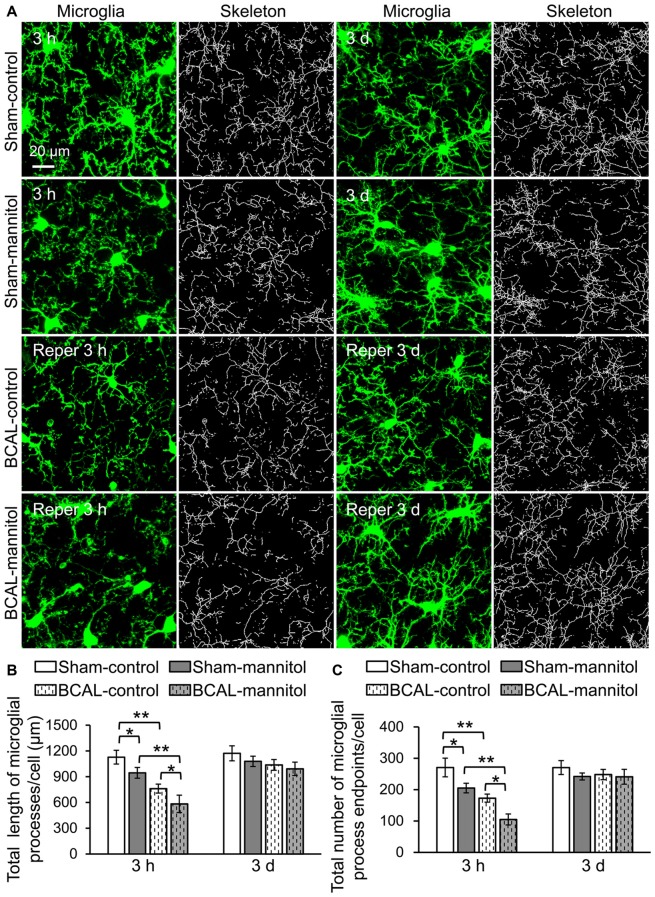
Mannitol-induced rapid morphological change of microglia following reperfusion after transient global cerebral ischemia. **(A)** Representative confocal images of microglia (green) and skeletonized microglia in sham-control, sham-mannitol, BCAL-control and BCAL-mannitol mice at 3 h and 3 days after ischemia. **(B,C)** Total length and total number of microglial processes in different groups, respectively. The total length of microglial process per cell and total number of microglial process endpoints at 3 h were significantly reduced after ischemia or mannitol treatment alone, and mannitol treatment in ischemic mice caused further decrease in the total length or total number of microglial process endpoints at 3 h (*n* = 6 mice, each group. *p < 0.05 and **p < 0.01). However, there are no significant differences in total length of microglial processes per cell or total number of microglial process endpoints per cell in different groups at 3 days (*n* = 6 mice, each group. *p* > 0.05).

Microglia are sensitive to cerebral microenvironment and can respond to a variety of environmental stimuli or pathological changes (Keren-Shaul et al., 2017). To investigate the morphological changes of microglia in response to extravasation, we analyzed microglial morphology with skeletonization analysis using ImageJ software (Morrison and Filosa, 2013). The total number of microglial process endpoints and total length of microglial processes of each cell were quantified to assess the complexity of microglial morphology (Figure 2A). In sham-operated mice, the total number of microglial process endpoints and the total length of microglial processes of each cell were significantly lower than that of sham-control mice at 3 h after mannitol injection. However, there were no significant differences in microglial morphology at 3 days between sham-control and sham-mannitol mice. We then evaluated the effect of ischemia on microglial morphology. In BCAL-control mice, the total number of microglial process endpoints and the total length of microglial processes of each cell were significantly reduced compared with that of sham-control mice at 3 h after reperfusion, but these differences are not significant at 3 days after reperfusion (Figures 2B,C), suggesting that the effect of leaked BBB on microglial morphology only exists in the early stage of reperfusion after ischemia. We further evaluated the effect of mannitol on microglial morphology in ischemic mice. Remarkably, both the total number of microglial process endpoints and total length of microglial processes in BCAL-mannitol mice were further reduced compared with that of BCAL-control mice at 3 h after reperfusion (Figures 2B,C; Total length of microglial processes/cell (μm): 3 h, BCAL-control 761.53 ± 52.04 μm vs. BCAL-mannitol 583.20 ± 101.31 μm; Total number of microglial process endpoints/cell: 3 h, BCAL-control 172.90 ± 12.87 vs. BCAL-mannitol 104.87 ± 49.53; *p < 0.05, respectively), but these differences are not significant at 3 days after reperfusion compared with that of BCAL-control mice or sham-mannitol mice.

**Figure 3:**
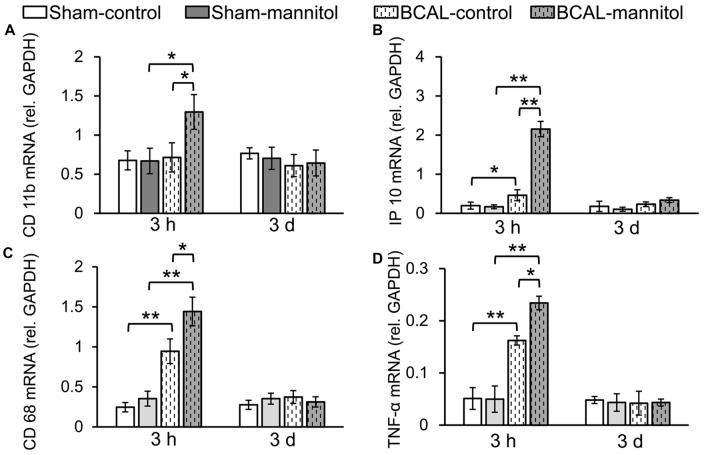
Mannitol-induced change in the mRNA expression level of inflammatory cytokine following reperfusion after ischemia. **(A–D)** There was no significant difference in mRNA expression levels of integrin alpha M *(CD11b), CD68*, tumor necrosis factor alpha *(TNF-α)* and chemokine (C-X-C motif) ligand 10 *(IP10)* at 3 h between sham-control mice and sham-mannitol mice. Ischemia-induced significant increase in *CD68, TNF-α* and *IP10*, but not *CD11b* at 3 h. However, the mRNA expression levels of *CD11b, CD68, TNF-α* and *IP10* are all significantly increased in BCAL-mannitol mice compared with BCAL-control mice or sham-mannitol mice at 3 h. There are no significant differences in the mRNA expression levels of *CD11b, CD68*, *TNF-α* and *IP10* at 3 days. *n* = 6 mice for each group. *p < 0.05 and **p < 0.01.

Microglia are monocyte-macrophage lineage cells, and the immune responses of microglia are tightly associated with inflammatory response (Jassam et al., 2017). To determine the inflammation response associated with BBB leakage, the mRNA expression of *CD11b, CD68, IP10* and *TNF-α* were detected via using fluorescent quantitative PCR. Quantitative analysis showed that there were no marked differences in mRNA expression level of *CD11b, CD68, IP10* and *TNF-α* between sham-control and sham-mannitol injected mice at both 3 h and 3 days (Figure 3). In BCAL-control mice, the mRNA expression levels of *CD68, IP10* and *TNF-α* increased significantly compared with that of sham-control mice at 3 h after reperfusion, but their expression levels returned to the sham-control level at 3 days after reperfusion (Figure 3). The expression level of *CD11b* showed no difference in BCAL-control mice compared with that of sham-control mice at 3 h and 3 days after reperfusion (Figure 3). However, in BCAL-mannitol mice, the expression levels of *CD11b, CD68, IP10* and *TNF-α* were all significantly higher than that of BCAL-control mice at 3 h after reperfusion, but their expression level also returned to control level at 3 days after reperfusion.

### Effect of Compromised BBB on Dendritic Structures

**Figure 4:**
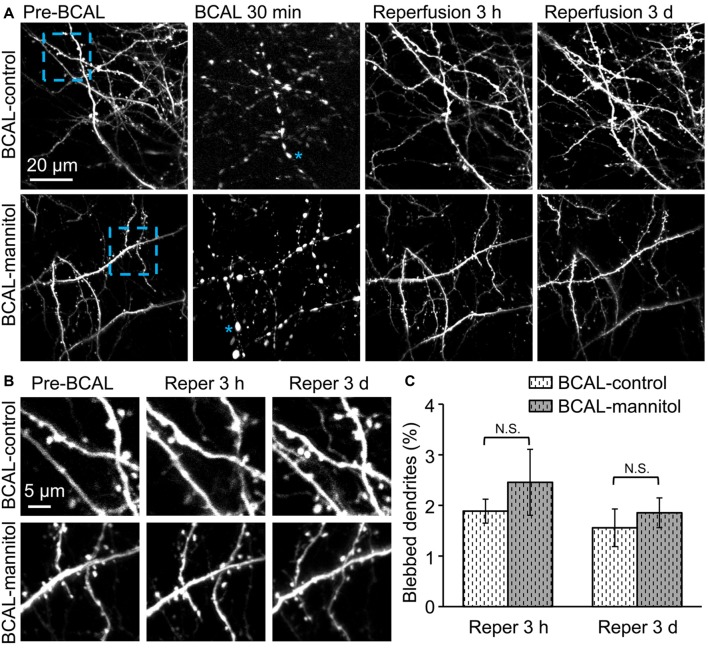
Mannitol did not affect the reversible recovery of the dendritic structure following global cerebral ischemia/reperfusion. **(A)** Morphological changes in dendritic structures before ischemia, 30 min after ischemia, 3 h and 3 days after reperfusion. Note that most of the blebbed dendrites recovered at 3 h after reperfusion. Asterisk (*) indicates blebbed dendrite. **(B)** Magnified view of the blue-boxed region in **(A)** showing dendritic structures before ischemia, at 3 h and 3 days after reperfusion. Note that mannitol-induced extravasation did not affect the reversible recovery of the dendritic structures. **(C)** Quantification of the percentage of blebbed dendrites in ischemic animals treated with mannitol or saline at 3 h and 3 days after reperfusion (*n* = 6 mice, each group). Note that there is no significant difference in dendritic beading in mannitol-injected mice and saline-injected mice. N.S., *p* > 0.05.

Time-lapse imaging indicated that there was no significant dendritic structural damage in sham-control and sham-mannitol mice (Figure 1B), suggesting that mannitol-induced extravasation did not have significant impact on dendritic structures in healthy animals. We further determined whether extravasation could affect the recovery of dendritic structure by evaluating the percentage of blebbed dendrites in BCAL-injured mice. Live imaging revealed that in BCAL-control and BCAL-mannitol mice, dendritic structures became beaded during 30 min of ischemia, but majority of beaded dendrites were restored after reperfusion and there was no significant difference of recovered dendrites between saline and mannitol injected mice at 3 h or 3 days (Figure 4). These results suggest that extravasation does not have significant impact on the reversible recovery of dendritic structures.

**Figure 5:**
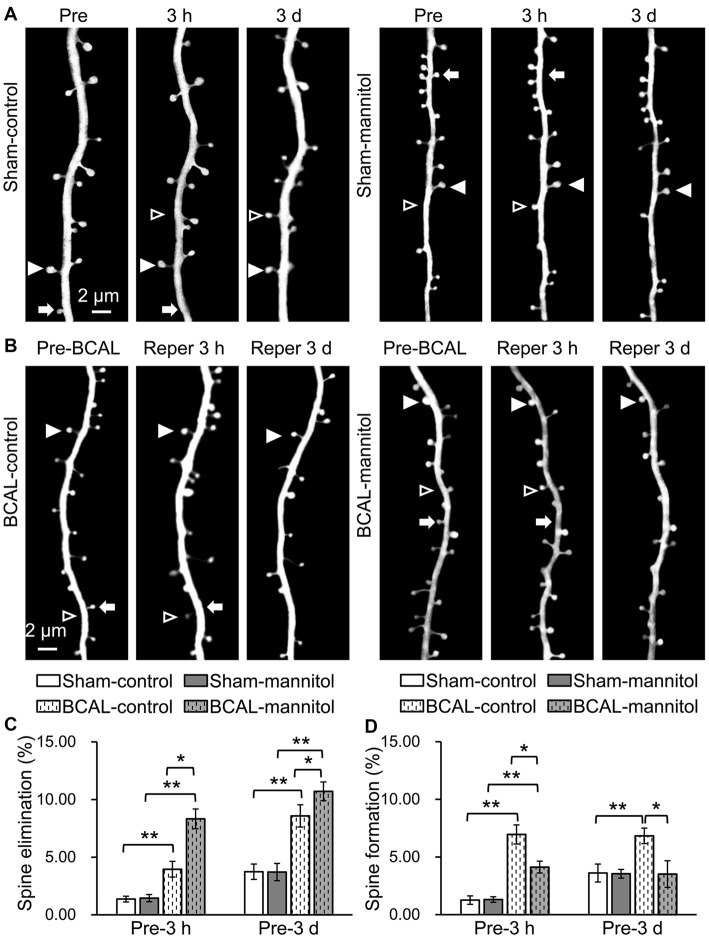
Mannitol-induced changes in spine formation and elimination following reperfusion after transient global cerebral ischemia. **(A,B)** Time-lapse imaging of dendritic structures showing spine formation or elimination in sham-control mice, sham-mannitol mice, BCAL-control mice and BCAL-mannitol mice. Open arrowheads, arrows and filled arrowheads indicate newly formed spines, eliminated spines and stable spines, respectively. **(C)** Spine elimination rate and **(D)** spine formation rate were significantly higher in BCAL-control than that of sham-control mice at 3 h and 3 days after reperfusion (*n* = 6 mice, each group. *p < 0.05 and **p < 0.01) and there were no significant changes in spine elimination rate and spine formation rate between sham-control mice and sham-mannitol mice at 3 h and 3 days (*n* = 6 mice, each group. *p* > 0.05). Note that spine elimination rate is significantly higher, but formation rate is significantly lower in BCAL-mannitol mice than that in BCAL-control mice at both 3 h and 3 days after reperfusion (*n* = 6 mice, each group. *p < 0.05 and **p < 0.01).

To further investigate the effect of extravasation on the plasticity of dendritic spines after acute global cerebral ischemia, we assessed the formation and elimination of spines. We first assessed the effect of mannitol-induced extravasation on both the formation and elimination rate of dendritic spines in sham-operated mice. Live imaging revealed that there were no significant differences in spine elimination rate and formation rate at both 3 h and 3 days between sham-control and sham-mannitol mice (Figures 5A,C,D; Pre-3 h, elimination rate: sham-mannitol 1.45 ± 0.31% vs. sham-control 1.36 ± 0.25%; Pre-3 h, formation rate: sham-mannitol 1.31 ± 0.24% vs. sham-control 1.26 ± 0.37%; Pre-3 days, elimination rate: sham-mannitol 3.71 ± 0.75% vs. sham-control 3.74 ± 0.67%; Pre-3 days, formation rate: sham-mannitol 3.55 ± 0.38% vs. sham-control 3.61 ± 0.78%). We further evaluated the effect of enhanced extravasation on the formation and elimination of dendritic spines in ischemia mice. Consistent with our previous findings, our data indicated that ischemia induced a rapid change in spine formation and elimination (Zhu et al., 2017b). In BCAL-control mice, both the elimination rate and formation rate were significantly higher than that of sham-control mice at both 3 h and 3 days after reperfusion, respectively (Figures 5B–D; Pre-3 h, BCAL-control, elimination rate 3.95 ± 0.68%, formation rate 6.96 ± 0.83%; Pre-3 days, BCAL-control, elimination rate 8.58 ± 0.96%, formation rate 6.83 ± 0.67%; **p < 0.01, respectively). Importantly, the spine elimination rate in BCAL-mannitol mice was even higher than BCAL-control mice, but the formation rate was significantly lower than the BCAL-control mice at both 3 h and 3 days after reperfusion (Figures 5C,D; Pre-3 h, BCAL-mannitol, elimination rate 8.32 ± 0.86%, formation rate 4.12 ± 0.51%; Pre-3 days, BCAL-mannitol, elimination rate 10.71 ± 0.81%, formation rate 3.52 ± 1.16%; *p < 0.05, **p < 0.01, respectively). Notably, the elimination rate was much higher than the formation rate in BCAL-mannitol mice, suggesting there was an increased loss of dendritic spines at both 3 h and 3 days after reperfusion. In conclusion, these data suggest that increased BBB permeability induced by mannitol has no significant effect on dendritic spines in healthy mice but may induce excessive spine loss following reperfusion after global cerebral ischemia.

**Figure 6:**
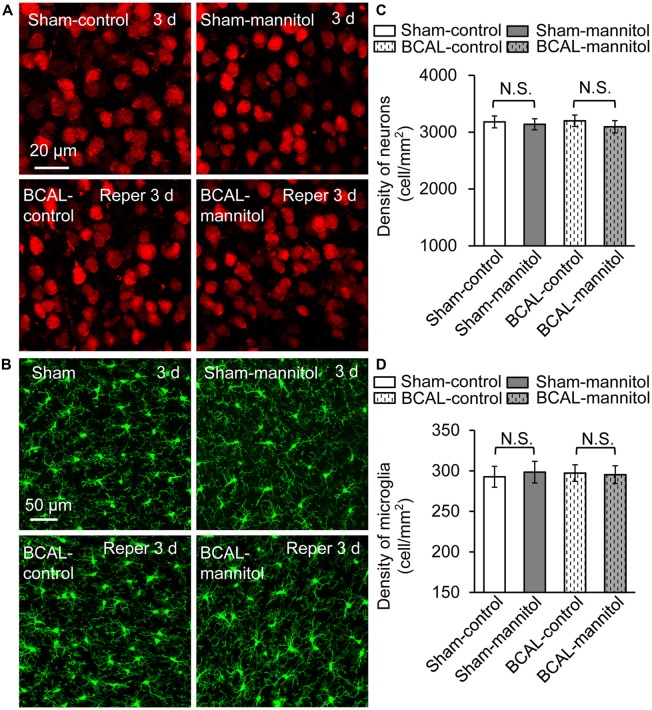
Mannitol did not affect the densities of neurons or microglia after ischemia. **(A)** Representative confocal images of the neurons (red) in different groups. **(B)** Confocal images showing the morphology of microglia (green) in different groups. **(C)** Quantification of neuronal density showing that there is no significant difference in different groups (*n* = 6 mice, each group. N.S., *p* > 0.05). **(D)** The density of microglia was not changed after transient ischemia and/or mannitol treatment (*n* = 6 mice, each group. N.S., *p* > 0.05).

To verify whether extravasation can affect the survival of neurons and microglia after 30 min of global ischemia, we quantified the densities of neurons and microglia in fixed brain tissue. For both sham mice and BCAL-injured mice, the densities of neurons and microglia showed no significant differences at 3 days (Figure 6). Therefore, mannitol-induced extravasation does not impact the densities of neurons and microglia after transient global ischemia. To investigate the activation of astrocyte in response to extravasation, we used glial fibrillary acidic protein (GFAP) staining in fixed brain tissue to examine the response of cortical astrocytes. The results showed that there was no significant activation of astrocyte in the cortices of all the four groups of animals (Sham-control, Sham-mannitol, BCAL-control, and BCAL-mannitol; Supplementary Figure S2) at 3 days after transient ischemia, suggesting that neither the transient global ischemia nor the mannitol treatment induces the activation of astrocyte in the cortices. To verify whether extravasation can affect the brain edema, we quantified the water content of the brain tissues at 3 days after 30 min of transient global ischemia. For both sham mice (Sham-control, Sham-mannitol) and BCAL-injured mice (BCAL-control, BCAL-mannitol), the water content showed no significant differences (Supplementary Figure S1B). These data suggested that cerebral edema was not induced in this transient global ischemia model, and mannitol does not impact water content of brain tissues in this case.

## Discussion

The BBB can ensure the ample supply of oxygen and glucose to the brain as well as maintaining a healthy microenvironment of cerebrum (Joó, 1987; Betsholtz, 2014). However, the integrity of BBB can be disrupted rapidly after ischemic stroke (Park et al., 2014; Shi et al., 2017). In the present research, we evaluated the impact of increased BBB permeability on microglial activation and dendritic structures. Microglia can retract their processes and change their gene expression in response to ischemia (Masuda et al., 2011; Morrison and Filosa, 2013). In this study, we observed a marked microglial de-ramification after mannitol treatment in the sham-operated mice, but there was no significant difference in mRNA expression level of inflammatory cytokines, suggesting that morphological change of microglia occurs earlier than the inflammatory response after BBB leakage. These data are in accordance with previous findings that the de-ramification of microglia is a consequence in response to the BBB leakage in CD200 deficient mice (Denieffe et al., 2013), and the expression level of inflammatory factors are low throughout the early stage (within 60 min) of BBB leakage (Sil et al., 2016). However, in BCAL-mannitol mice, we observed an enhanced de-ramification of microglia at 3 h after reperfusion compared to the BCAL-control animals, and the expression level of inflammatory cytokines was also significantly increased at 3 h after reperfusion, indicating that immunological response can be greatly enhanced by blood extravasation in ischemic mice.

In ischemia-operated mice, the data indicated that the reversible recovery of dendritic structures was not affected by mannitol treatment during reperfusion, suggesting that the change in BBB permeability is a minor factor for the integrity of neural network compared to local blood flow (Zhu et al., 2017b). In addition, in sham-operated mice, our results showed that mannitol-induced acute BBB leakage did not have significant impact on dendritic structures and spine plasticity, implying that extravasation alone has little acute effect on the neuronal structures and remodeling of cortical circuits. These data is consistent with previous findings that there is little acute collateral damage of dendrites by BBB extravasation during the first few hours after ischemic stroke (Zhang and Murphy, 2007; Zhu et al., 2017b). Previous study has shown that chronic BBB breakdown can induce dendritic spine loss over months in pericyte-deficient transgenic mice (Bell et al., 2010). Whether extravasation has long-term effect on neuronal structures or stability of cortical circuits remains to be evaluated further in future study. In addition, here we evaluated the impact of extravasation using a transient ischemia model, and the impact of excessive extravasation on dendritic structures in other stroke models remains to be determined.

Although increased BBB permeability did not change the reversible recovery of dendritic structures after ischemia, intravital imaging showed that increased BBB permeability exacerbated the loss of dendritic spines when layered on top of ischemia, indicating that extravasation can increase the vulnerability of synaptic structure to ischemia. Our study is in agreement with a previous study that increased BBB permeability exacerbates synaptic damage and promotes the loss of dendritic structure in peri-infarct regions where the BBB disruption occurs after ischemic stroke in diabetic mice (Reeson et al., 2015). Dendritic spine is the major postsynaptic targets in the CNS (Nishiyama and Yasuda, 2015). Spine dynamics are considered to be crucial for neural development and circuit plasticity under normal physical conditions (Grutzendler et al., 2002; Zuo et al., 2005; Bhatt et al., 2009; Nishiyama and Yasuda, 2015), and can be regulated by motor learning and novel sensory experience (Yang et al., 2009, 2014; Ashby and Isaac, 2011; Huang and Yang, 2015). However, the dynamics of dendritic spines can be changed by ischemia and other pathological insults (Hasbani et al., 2001; Heiss et al., 2017; Suresh and Dunaevsky, 2017; Zhu et al., 2017b). Previous studies indicate that ischemic stroke can induce extensive increase in spine formation and elimination (Brown et al., 2007; Zhu et al., 2017b). The increased spine formation and elimination rate after ischemia is suggested to be associated with the rewiring of neuronal circuits and enhanced plasticity can contribute to the recovery of cortical function (Dijkhuizen et al., 2001; Ward and Cohen, 2004; Murphy and Corbett, 2009). In our present study, live imaging data showed the increased BBB leakage aggravated the loss of dendritic spines, suggesting that extravasation may have accumulating and detrimental effect on the restoration of cortical network following ischemia.

As resident myeloid cells of the CNS, microglia have been suggested to be related not only to the immune response but also to the synaptic plasticity. Evidences in the literature reveal that dendritic dynamics are associated with microglial activation (Stephan et al., 2012; Kettenmann et al., 2013). Studies have shown that the microglia can eliminate dendritic spines by removing the synaptic button via phagocytosis (Kettenmann et al., 2013), and the learning-dependent dendritic spines formation is regulated by microglia via BDNF signaling in postnatal period (Paolicelli et al., 2011; Parkhurst et al., 2013). In addition, microglia can also employ a diverse repertoire of proteins in the innate immune system or complement pathways and play an important role in the removal of synapses (Stephan et al., 2013). Our data is consistent with previous studies that activated microglia can contribute to the formation and elimination of synapses during ischemia/reperfusion (Bruce-Keller, 1999; Wake et al., 2009). Although our present study revealed that microglial activation is associated with spine dynamics, the detailed mechanism underlying the microglial activation and synaptic plasticity following ischemia remains to be determined in future study.

Our results indicate that increased BBB extravasation is accompanied with an increased expression of inflammatory cytokines *CD11b, CD68, IP10* and *TNF-α* at 3 h after reperfusion in BCAL-mannitol mice. As key mediators of pathological response, inflammatory cytokines are closely associated with synaptic plasticity (Yang et al., 2013). Previous studies have shown that inflammatory cytokines play a key role in regulating synaptic plasticity in major depressive disorder (Khairova et al., 2009), and inflammation can trigger the alteration and degeneration of synapses in autoimmune encephalomyelitis (Centonze et al., 2009). Our present study indicates that enhanced expression of inflammatory cytokines may contribute to the increased loss of dendritic spines when layered on the top of ischemia.

Mannitol, as a sugar alcohol, is widely used in many pathological models and clinical therapy to reduce cerebral edema in acute intracerebral hemorrhage or to mediate the permeation of macromolecular drugs/stem cells through BBB (Borlongan et al., 2004; Stonestreet et al., 2012; Wang et al., 2015). However, there are still some controversies about the effect of mannitol in stroke treatment (Bereczki et al., 2003; Wang et al., 2015). Here we showed that mannitol did not cause significant changes in spine formation nor in spine elimination in sham-operated mice. Nevertheless, our data suggested that excessive extravasation induced by mannitol following ischemia caused significant loss of dendritic spines. Thus, using mannitol as a clinical therapy in ischemic patients should be cautiously evaluated.

In summary, the data from present study suggested that although mannitol-induced extravasation of blood has little acute effect on inflammatory response and overall dendritic structures in sham-operated mice, it causes a significant microglial activation, inflammatory response and loss of dendrite spines after ischemia. Although mannitol is widely used to reduce edema formation in the brain in clinical situations, increased BBB permeability associated with mannitol treatment may have a potential clinical risk following ischemic stroke, and strategies on controlling excessive extravasation in stroke patients may have therapeutic potentials for the treatment of stroke.

## Author Contributions

FJ, YR and SZ designed the research plan. FJ, HG, YR, LZ, XC and XX performed the experiments. FJ, YR, LZ, XC and ZY analyzed the data. FJ, YR, HG and SZ wrote the article.

## Conflict of Interest Statement

The authors declare that the research was conducted in the absence of any commercial or financial relationships that could be construed as a potential conflict of interest.

## Footnotes

^1^http://rsb.info.nih.gov/ij/

^2^http://imagejdocu.tudor.lu

## Abbreviations

AD: Alzheimer’s disease
BBB: Blood-brain barrier
BCAL: Bilateral common carotid artery ligation
*CD11b*: Integrin alpha M
CNS: Central nervous system
EB: Evans blue
*GAPDH*: Glyceraldehyde 3-phosphate dehydrogenase
GFAP: Glial fibrillary acidic protein
GFP: Green fluorescent proteins
*IP10*: Chemokine (C-X-C motif) ligand 10
MCAO: Middle cerebral artery occlusion
PBS: Phosphate-buffered saline
PFA: Paraformaldehyde
ROI: Region of interest
*TNF-α*: Tumor necrosis factor alpha
YFP: Yellow fluorescent proteins.

## References

[B1] Ashby M. C. Isaac J. T. (2011). Maturation of a recurrent excitatory neocortical circuit by experience-dependent unsilencing of newly formed dendritic spines. Neuron 70, 510–521. 10.1016/j.neuron.2011.02.057 21555076 PMC3092126

[B2] Bell R. D. Winkler E. A. Sagare A. P. Singh I. LaRue B. Deane R.. (2010). Pericytes control key neurovascular functions and neuronal phenotype in the adult brain and during brain aging. Neuron 68, 409–427. 10.1016/j.neuron.2010.09.043 21040844 PMC3056408

[B3] Bereczki D. Mihálka L. Szatmári S. Fekete K. Di Cesar D. Fülesdi B.. (2003). Mannitol use in acute stroke: case fatality at 30 days and 1 year. Stroke 34, 1730–1735. 10.1161/01.str.0000078658.52316.e8 12817105

[B4] Betsholtz C. (2014). Physiology: double function at the blood-brain barrier. Nature 509, 432–433. 10.1038/nature13339 24828036

[B5] Bhatt D. H. Zhang S. Gan W. B. (2009). Dendritic spine dynamics. Annu. Rev. Physiol. 71, 261–282. 10.1146/annurev.physiol.010908.163140 19575680

[B6] Borlongan C. V. Glover L. E. Sanberg P. R. Hess D. C. (2012). Permeating the blood brain barrier and abrogating the inflammation in stroke: implications for stroke therapy. Curr. Pharm. Des. 18, 3670–3676. 10.2174/138161212802002841 22574981 PMC3411920

[B7] Borlongan C. V. Hadman M. Sanberg C. D. Sanberg P. R. (2004). Central nervous system entry of peripherally injected umbilical cord blood cells is not required for neuroprotection in stroke. Stroke 35, 2385–2389. 10.1161/01.STR.0000141680.49960.d7 15345799

[B8] Brown C. E. Li P. Boyd J. D. Delaney K. R. Murphy T. H. (2007). Extensive turnover of dendritic spines and vascular remodeling in cortical tissues recovering from stroke. J. Neurosci. 27, 4101–4109. 10.1523/jneurosci.4295-06.2007 17428988 PMC6672555

[B9] Bruce-Keller A. J. (1999). Microglial-neuronal interactions in synaptic damage and recovery. J. Neurosci. Res. 58, 191–201. 10.1002/(SICI)1097-4547(19991001)58:1<191::AID-JNR17>3.0.CO;2-E 10491582

[B10] Centonze D. Muzio L. Rossi S. Cavasinni F. De Chiara V. Bergami A.. (2009). Inflammation triggers synaptic alteration and degeneration in experimental autoimmune encephalomyelitis. J. Neurosci. 29, 3442–3452. 10.1523/jneurosci.5804-08.2009 19295150 PMC6665268

[B11] Chen J. Ning R. Zacharek A. Cui C. Cui X. Yan T.. (2016). MiR-126 contributes to human umbilical cord blood cell-induced neurorestorative effects after stroke in type-2 diabetic mice. Stem Cells 34, 102–113. 10.1002/stem.2193 26299579 PMC4713352

[B12] Choi K. H. Kim H. S. Park M. S. Kim J. T. Kim J. H. Cho K. A.. (2016). Regulation of caveolin-1 expression determines early brain edema after experimental focal cerebral ischemia. Stroke 47, 1336–1343. 10.1161/strokeaha.116.013205 27012742

[B13] Denieffe S. Kelly R. J. McDonald C. Lyons A. Lynch M. A. (2013). Classical activation of microglia in CD200-deficient mice is a consequence of blood brain barrier permeability and infiltration of peripheral cells. Brain Behav. Immun. 34, 86–97. 10.1016/j.bbi.2013.07.174 23916893

[B14] Dijkhuizen R. M. Ren J. M. Mandeville J. B. Wu O. Ozdag F. M. Moskowitz M. A.. (2001). Functional magnetic resonance imaging of reorganization in rat brain after stroke. Proc. Natl. Acad. Sci. U S A 98, 12766–12771. 10.1073/pnas.231235598 11606760 PMC60128

[B15] Gelderblom M. Leypoldt F. Steinbach K. Behrens D. Choe C. U. Siler D. A.. (2009). Temporal and spatial dynamics of cerebral immune cell accumulation in stroke. Stroke 40, 1849–1857. 10.1161/strokeaha.108.534503 19265055

[B16] Grutzendler J. Kasthuri N. Gan W. B. (2002). Long-term dendritic spine stability in the adult cortex. Nature 420, 812–816. 10.1038/nature01276 12490949

[B17] Hasbani M. J. Schlief M. L. Fisher D. A. Goldberg M. P. (2001). Dendritic spines lost during glutamate receptor activation reemerge at original sites of synaptic contact. J. Neurosci. 21, 2393–2403. 10.1523/jneurosci.21-07-02393.2001 11264313 PMC6762381

[B18] Hawkins B. T. Davis T. P. (2005). The blood-brain barrier/neurovascular unit in health and disease. Pharmacol. Rev. 57, 173–185. 10.1124/pr.57.2.4 15914466

[B19] Heiss J. K. Barrett J. Yu Z. Z. Haas L. T. Kostylev M. A. Strittmatter S. M. (2017). Early activation of experience-independent dendritic spine turnover in a mouse model of Alzheimer’s disease. Cereb. Cortex 27, 3660–3674. 10.1093/cercor/bhw188 27365298 PMC6059166

[B20] Huang L. Y. Yang G. (2015). Repeated exposure to ketamine-xylazine during early development impairs motor learning-dependent dendritic spine plasticity in adulthood. Anesthesiology 122, 821–831. 10.1097/aln.0000000000000579 25575163 PMC4366292

[B21] Ishikawa H. Tajiri N. Shinozuka K. Vasconcellos J. Kaneko Y. Lee H. J.. (2013). Vasculogenesis in experimental stroke after human cerebral endothelial cell transplantation. Stroke 44, 3473–3481. 10.1161/strokeaha.113.001943 24130140 PMC4083631

[B22] Jassam Y. N. Izzy S. Whalen M. McGavern D. B. El Khoury J. (2017). Neuroimmunology of traumatic brain injury: time for a paradigm shift. Neuron 95, 1246–1265. 10.1016/j.neuron.2017.07.010 28910616 PMC5678753

[B23] Joó F. (1987). The blood-brain barrier. Nature 329:208 10.1385/1-59259-419-0:133 3627266

[B24] Joshi S. Ergin A. Wang M. Reif R. Zhang J. Bruce J. N.. (2011). Inconsistent blood brain barrier disruption by intraarterial mannitol in rabbits: implications for chemotherapy. J. Neurooncol. 104, 11–19. 10.1007/s11060-010-0466-4 21153681 PMC4013427

[B25] Keren-Shaul H. Spinrad A. Weiner A. Matcovitch-Natan O. Dvir-Szternfeld R. Ulland T. K.. (2017). A Unique microglia type associated with restricting development of Alzheimer’s disease. Cell 169, 1276.e17–1290.e17. 10.1016/j.cell.2017.05.018 28602351

[B26] Kettenmann H. Kirchhoff F. Verkhratsky A. (2013). Microglia: new roles for the synaptic stripper. Neuron 77, 10–18. 10.1016/j.neuron.2012.12.023 23312512

[B27] Khairova R. A. Machado-Vieira R. Du J. Manji H. K. (2009). A potential role for pro-inflammatory cytokines in regulating synaptic plasticity in major depressive disorder. Int. J. Neuropsychopharmacol. 12, 561–578. 10.1017/s1461145709009924 19224657 PMC2771334

[B28] Lin C. Y. Chang C. Cheung W. M. Lin M. H. Chen J. J. Hsu C. Y.. (2008). Dynamic changes in vascular permeability, cerebral blood volume, vascular density and size after transient focal cerebral ischemia in rats: evaluation with contrast-enhanced magnetic resonance imaging. J. Cereb. Blood Flow Metab. 28, 1491–1501. 10.1038/jcbfm.2008.42 18478021

[B29] Masuda T. Croom D. Hida H. Kirov S. A. (2011). Capillary blood flow around microglial somata determines dynamics of microglial processes in ischemic conditions. Glia 59, 1744–1753. 10.1002/glia.21220 21800362 PMC3174346

[B30] Moro M. A. Cardenas A. Hurtado O. Leza J. C. Lizasoain I. (2004). Role of nitric oxide after brain ischaemia. Cell Calcium 36, 265–275. 10.1016/j.ceca.2004.02.011 15261482

[B31] Morrison H. W. Filosa J. A. (2013). A quantitative spatiotemporal analysis of microglia morphology during ischemic stroke and reperfusion. J. Neuroinflammation 10:4. 10.1186/1742-2094-10-4 23311642 PMC3570327

[B32] Murphy T. H. Corbett D. (2009). Plasticity during stroke recovery: from synapse to behaviour. Nat. Rev. Neurosci. 10, 861–872. 10.1038/nrn2735 19888284

[B33] Nahirney P. C. Reeson P. Brown C. E. (2016). Ultrastructural analysis of blood-brain barrier breakdown in the peri-infarct zone in young adult and aged mice. J. Cereb. Blood Flow Metab. 36, 413–425. 10.1177/0271678X15608396 26661190 PMC4759675

[B34] Nishimura N. Schaffer C. B. Friedman B. Tsai P. S. Lyden P. D. Kleinfeld D. (2006). Targeted insult to subsurface cortical blood vessels using ultrashort laser pulses: three models of stroke. Nat. Methods 3, 99–108. 10.1038/nmeth844 16432519

[B35] Nishiyama J. Yasuda R. (2015). Biochemical computation for spine structural plasticity. Neuron 87, 63–75. 10.1016/j.neuron.2015.05.043 26139370 PMC4722820

[B36] Olmedo-Diaz S. Estévez-Silva H. Orädd G. Af Bjerkén S. Marcellino D. Virel A. (2017). An altered blood-brain barrier contributes to brain iron accumulation and neuroinflammation in the 6-OHDA rat model of Parkinson’s disease. Neuroscience 362, 141–151. 10.1016/j.neuroscience.2017.08.023 28842186

[B37] Olmos-Alonso A. Schetters S. T. Sri S. Askew K. Mancuso R. Vargas-Caballero M.. (2016). Pharmacological targeting of CSF1R inhibits microglial proliferation and prevents the progression of Alzheimer’s-like pathology. Brain 139, 891–907. 10.1093/brain/awv379 26747862 PMC4766375

[B38] Paolicelli R. C. Bolasco G. Pagani F. Maggi L. Scianni M. Panzanelli P.. (2011). Synaptic pruning by microglia is necessary for normal brain development. Science 333, 1456–1458. 10.1126/science.1202529 21778362

[B39] Park J. Y. Lee S. K. Kim J. Y. Je K. H. Schellingerhout D. Kim D. E. (2014). A new micro-computed tomography-based high-resolution blood-brain barrier imaging technique to study ischemic stroke. Stroke 45, 2480–2484. 10.1161/strokeaha.114.006297 25013021

[B40] Parkhurst C. N. Yang G. Ninan I. Savas J. N. Yates J. R. Lafaille J. J.. (2013). Microglia promote learning-dependent synapse formation through brain-derived neurotrophic factor. Cell 155, 1596–1609. 10.1016/j.cell.2013.11.030 24360280 PMC4033691

[B41] Reeson P. Tennant K. A. Gerrow K. Wang J. Weiser Novak S. Thompson K.. (2015). Delayed inhibition of VEGF signaling after stroke attenuates blood-brain barrier breakdown and improves functional recovery in a comorbidity-dependent manner. J. Neurosci. 35, 5128–5143. 10.1523/jneurosci.2810-14.2015 25834040 PMC6705411

[B42] Sandoval K. E. Witt K. A. (2008). Blood-brain barrier tight junction permeability and ischemic stroke. Neurobiol. Dis. 32, 200–219. 10.1016/j.nbd.2008.08.005 18790057

[B43] Shi S. Qi Z. Ma Q. Pan R. Timmins G. S. Zhao Y.. (2017). Normobaric hyperoxia reduces blood occludin fragments in rats and patients with acute ischemic stroke. Stroke 48, 2848–2854. 10.1161/strokeaha.117.017713 28931617 PMC5659343

[B44] Shi Y. Zhang L. Pu H. Mao L. Hu X. Jiang X.. (2016). Rapid endothelial cytoskeletal reorganization enables early blood-brain barrier disruption and long-term ischaemic reperfusion brain injury. Nat. Commun. 7:10523. 10.1038/ncomms10523 26813496 PMC4737895

[B45] Sil S. Ghosh A. Ghosh T. (2016). Impairment of blood brain barrier is related with the neuroinflammation induced peripheral immune status in intracerebroventricular colchicine injected rats: An experimental study with mannitol. Brain Res. 1646, 278–286. 10.1016/j.brainres.2016.05.052 27288705

[B46] Song X. Walczak P. He X. Yang X. Pearl M. Bulte J. W.. (2016). Salicylic acid analogues as chemical exchange saturation transfer MRI contrast agents for the assessment of brain perfusion territory and blood-brain barrier opening after intra-arterial infusion. J. Cereb. Blood Flow Metab. 36, 1186–1194. 10.1177/0271678x16637882 26980755 PMC4929703

[B47] Stephan A. H. Barres B. A. Stevens B. (2012). The complement system: an unexpected role in synaptic pruning during development and disease. Annu. Rev. Neurosci. 35, 369–389. 10.1146/annurev-neuro-061010-113810 22715882

[B48] Stephan A. H. Madison D. V. Mateos J. M. Fraser D. A. Lovelett E. A. Coutellier L.. (2013). A dramatic increase of C1q protein in the CNS during normal aging. J. Neurosci. 33, 13460–13474. 10.1523/jneurosci.1333-13.2013 23946404 PMC3742932

[B49] Stonestreet B. S. Sadowska G. B. Hanumara R. C. Petrache M. Petersson K. H. Patlak C. S. (2012). Comparative effects of glucose- and mannitol-induced osmolar stress on blood-brain barrier function in ovine fetuses and lambs. J. Cereb. Blood Flow Metab. 32, 115–126. 10.1038/jcbfm.2011.114 21878946 PMC3324288

[B50] Suresh A. Dunaevsky A. (2017). Relationship between synaptic AMPAR and spine dynamics: impairments in the FXS mouse. Cereb. Cortex 27, 4244–4256. 10.1093/cercor/bhx128 28541473 PMC6057510

[B51] Ulrich J. D. Huynh T. P. Holtzman D. M. (2015). Re-evaluation of the blood-brain barrier in the presence of Alzheimer’s disease pathology. Neuron 88, 237–239. 10.1016/j.neuron.2015.10.008 26494271

[B52] Wake H. Moorhouse A. J. Jinno S. Kohsaka S. Nabekura J. (2009). Resting microglia directly monitor the functional state of synapses *in vivo* and determine the fate of ischemic terminals. J. Neurosci. 29, 3974–3980. 10.1523/jneurosci.4363-08.2009 19339593 PMC6665392

[B53] Wang X. Arima H. Yang J. Zhang S. Wu G. Woodward M.. (2015). Mannitol and outcome in intracerebral hemorrhage: propensity score and multivariable intensive blood pressure reduction in acute cerebral hemorrhage trial 2 results. Stroke 46, 2762–2767. 10.1161/strokeaha.115.009357 26265125

[B54] Ward N. S. Cohen L. G. (2004). Mechanisms underlying recovery of motor function after stroke. Arch. Neurol. 61, 1844–1848. 10.1001/archneur.61.12.1844 15596603 PMC3713312

[B55] Yang G. Lai C. S. Cichon J. Ma L. Li W. Gan W. B. (2014). Sleep promotes branch-specific formation of dendritic spines after learning. Science 344, 1173–1178. 10.1126/science.1249098 24904169 PMC4447313

[B56] Yang G. Pan F. Gan W. B. (2009). Stably maintained dendritic spines are associated with lifelong memories. Nature 462, 920–924. 10.1038/nature08577 19946265 PMC4724802

[B57] Yang G. Pan F. Parkhurst C. N. Grutzendler J. Gan W. B. (2010). Thinned-skull cranial window technique for long-term imaging of the cortex in live mice. Nat. Protoc. 5, 201–208. 10.1038/nprot.2009.222 20134419 PMC4690457

[B58] Yang G. Parkhurst C. N. Hayes S. Gan W. B. (2013). Peripheral elevation of TNF-α leads to early synaptic abnormalities in the mouse somatosensory cortex in experimental autoimmune encephalomyelitis. Proc. Natl. Acad. Sci. U S A 110, 10306–10311. 10.1073/pnas.1222895110 23733958 PMC3690863

[B59] Zhang H. Park J. H. Maharjan S. Park J. A. Choi K. S. Park H.. (2017). Sac-1004, a vascular leakage blocker, reduces cerebral ischemia-reperfusion injury by suppressing blood-brain barrier disruption and inflammation. J. Neuroinflammation 14:122. 10.1186/s12974-017-0897-3 28645333 PMC5481915

[B60] Zhang S. Murphy T. H. (2007). Imaging the impact of cortical microcirculation on synaptic structure and sensory-evoked hemodynamic responses *in vivo*. PLoS Biol. 5:e119. 10.1371/journal.pbio.0050119 17456007 PMC1854912

[B61] Zhao Z. Nelson A. R. Betsholtz C. Zlokovic B. V. (2015). Establishment and dysfunction of the blood-brain barrier. Cell 163, 1064–1078. 10.1016/j.cell.2015.10.067 26590417 PMC4655822

[B62] Zhu L. Wang L. Ju F. Khan A. Cheng X. Zhang S. (2017a). Reversible recovery of neuronal structures depends on the degree of neuronal damage after global cerebral ischemia in mice. Exp. Neurol. 289, 1–8. 10.1016/j.expneurol.2016.12.002 27940018

[B63] Zhu L. Wang L. Ju F. Ran Y. Wang C. Zhang S. (2017b). Transient global cerebral ischemia induces rapid and sustained reorganization of synaptic structures. J. Cereb. Blood Flow Metab. 37, 2756–2767. 10.1177/0271678x16674736 27798269 PMC5536786

[B64] Zlokovic B. V. (2008). The blood-brain barrier in health and chronic neurodegenerative disorders. Neuron 57, 178–201. 10.1016/j.neuron.2008.01.003 18215617

[B65] Zuo Y. Lin A. Chang P. Gan W. B. (2005). Development of long-term dendritic spine stability in diverse regions of cerebral cortex. Neuron 46, 181–189. 10.1016/j.neuron.2005.04.001 15848798

