# Supplementary material for: Increased BBB Permeability Enhances Activation of Microglia and Exacerbates Loss of Dendritic Spines After Transient Global Cerebral Ischemia
Source: Front Cell Neurosci. 2018 Aug 3;12:236. doi: 10.3389/fncel.2018.00236 (PMC6085918; doi:10.3389/fncel.2018.00236)
Supplement: Supplementary file 1 [file Data_Sheet_1.docx]

Supplementary Material

Increased BBB permeability enhances activation of microglia and exacerbates loss of dendritic spines after transient global cerebral ischemia

**Furong Ju^1^, Yanli Ran^1^, Lirui Zhu^1^, Xiaofeng Cheng^1^, Hao Gao^1^, Xiaoxia Xi^2^, Zhanli Yang^1^, Shengxiang Zhang^1^***

*** Correspondence:** Shengxiang Zhang, Ph.D.: [sxzhang@lzu.edu.cn](mailto:sxzhang@lzu.edu.cn)

# Supplementary Methods and Figures

## Supplementary Methods

### 1.1.1 Immunofluoroscent labeling

Brain tissues fixed by 4% paraformaldehyde (PFA) were washed in phosphate buffered saline (PBS), and sectioned (30 μm) on a vibrating microtome (Leica). Sections were blocked with 10% goat serum dissolved in PBS for 30 min and then incubated with GFAP antibodies (1:200, Rabbit, Millipore) diluted in a buffer consisted of 0.01% Triton X-100 and 5% goat serum for 12 hours at 4°C. The sections were washed and then incubated with a secondary antibody (1:500, Goat anti-Rabbit TRITC, ZSGB-BIO) at room temperature.

### 1.1.2 Measurement of the brain water content

The wet weight of the brain tissue was measured after the tissue distraction. Then the tissue was dried in a 110℃ oven for 48 hours and the dry weight was measured. The water content θ was defined as: θ = (W_wet_ – W_dry_)/W_wet_ * 100%

## Supplementary Figures


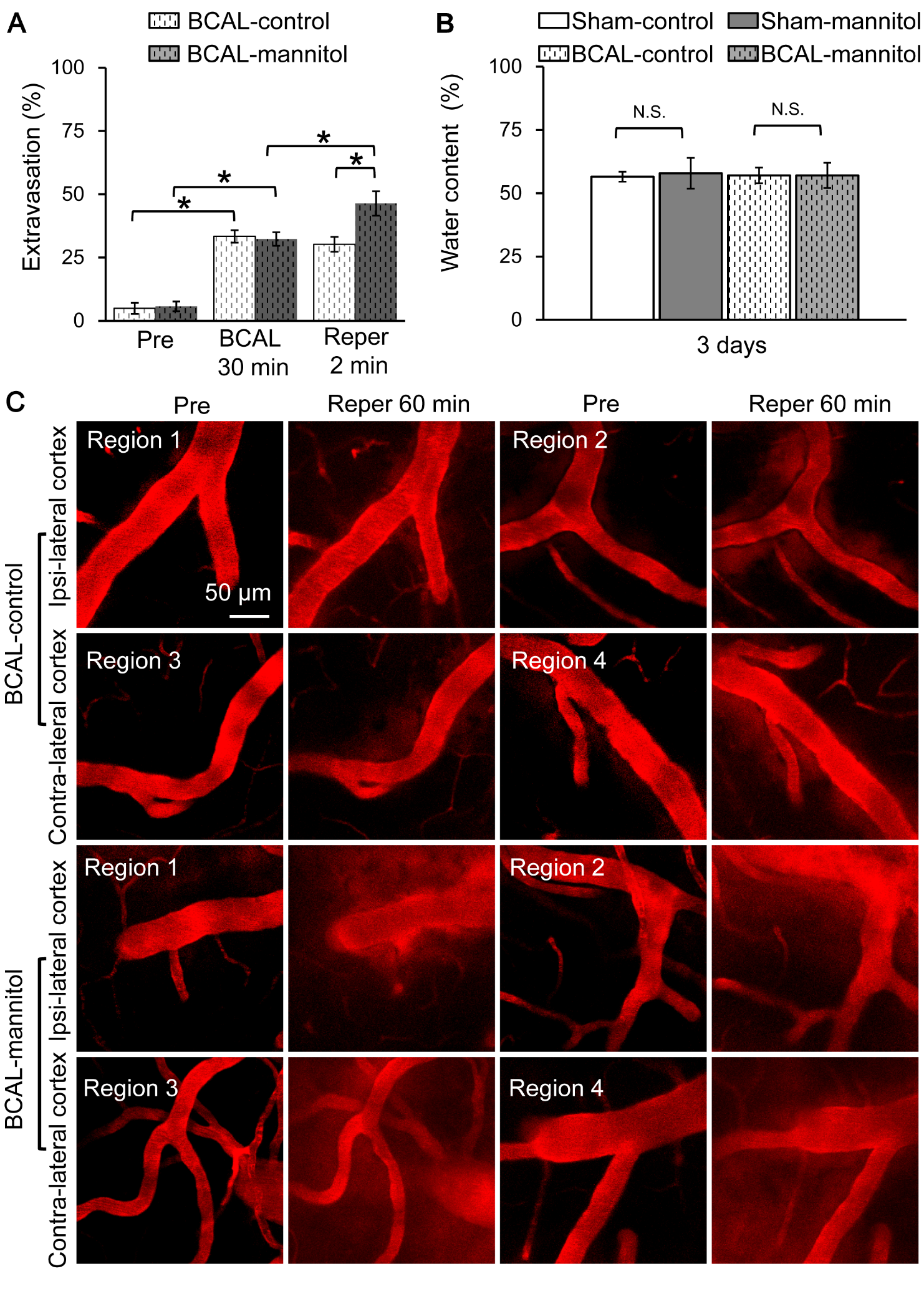


**Supplementary Figure 1.** BBB permeability and brain edema after transient ischemic stroke. **A.** Change in BBB permeability during ischemia and after mannitol infusion. Ischemia alone induced a significant increase in BBB permeability, and mannitol infusion further enhanced the leakage of blood plasma (**p*<0.05). **B.** Transient ischemia and mannitol treatment did not have significant effect on brain water content (*p*> 0.05). **C.** Intravital two-photon imaging of BBB leakage in four different cortical regions (two on each side). Significant extravasation was observed on both sides of the cerebral hemisphere during reperfusion after ischemia.


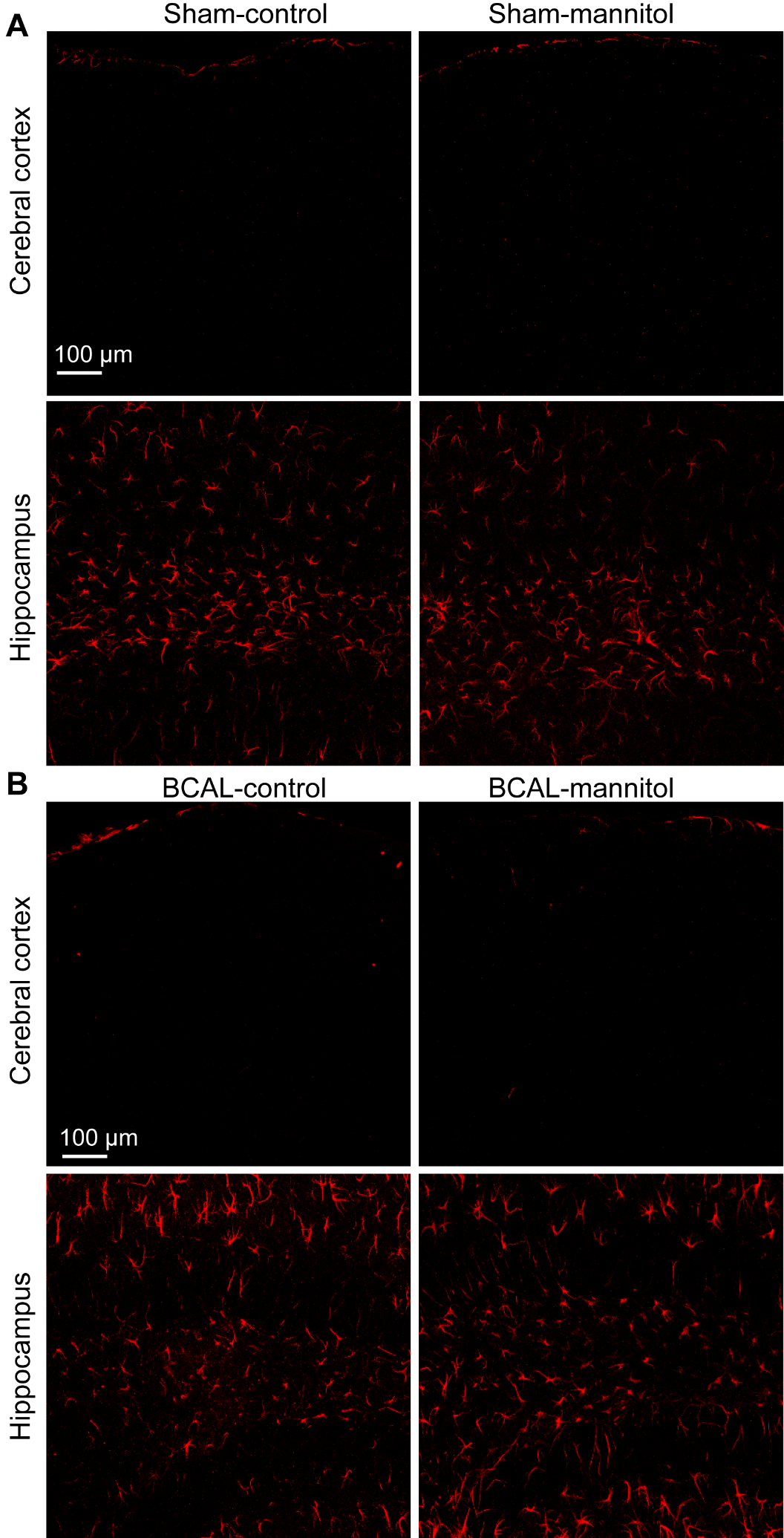


**Supplementary Figure 2.** Immunofluorescence staining of astrocytes after transient ischemic stroke. **A.** GFAP staining of astrocytes in sham animals (Sham-control and Sham-mannitol) after transient ischemic stroke. **B.** GFAP staining of astrocytes in ischemic animals (BCAL-control, and BCAL-mannitol) after transient ischemic stroke. GFAP positive cells were not detected in the cortices of all the four groups of mice (Sham-control, Sham-mannitol, BCAL-control, and BCAL-mannitol). GFAP staining in the hippocampus was used as a positive control. The results indicated that neither the transient global ischemia nor the mannitol treatment induced the activation of astrocyte in the cortices.
